# Optical Coherence Tomography Assessment of Macular Thickness in Alzheimer’s Dementia with Different Neuropsychological Severities

**DOI:** 10.3390/jpm13071118

**Published:** 2023-07-10

**Authors:** Chia-Chen Kao, Hui-Min Hsieh, Yo-Chen Chang, Hui-Chen Chu, Yuan-Han Yang, Shwu-Jiuan Sheu

**Affiliations:** 1 Department of Ophthalmology, Kaohsiung Medical University Hospital, Kaohsiung 80756, Taiwan; hsuanmigreen@gmail.com (C.-C.K.); ycchang.oph@gmail.com (Y.-C.C.); chuchuchen25@gmail.com (H.-C.C.); 2 Department of Ophthalmology, Kaohsiung Medical University, Kaohsiung 807, Taiwan; 3 Department of Public Health, Kaohsiung Medical University, Kaohsiung 807, Taiwan; hsiehhm@gmail.com; 4 Department of Medical Research, Kaohsiung Medical University Hospital, Kaohsiung 80756, Taiwan; 5 Department of Community Medicine, Kaohsiung Medical University Hospital, Kaohsiung 80756, Taiwan; 6 Center for Big Data Research, Kaohsiung Medical University, Kaohsiung 807, Taiwan; 7 Research Center for Precision Environmental Medicine, Kaohsiung Medical University, Kaohsiung 807, Taiwan; 8 Department of Ophthalmology, Kaohsiung Municipal Siaogang Hospital, Kaohsiung 812, Taiwan; 9 Department of Neurology, Kaohsiung Medical University Hospital, Kaohsiung Medical University, Kaohsiung 807, Taiwan; 10 Neuroscience Research Center, Kaohsiung Medical University, Kaohsiung 812, Taiwan; 11 Department of Neurology, Kaohsiung Municipal Ta-Tung Hospital, Kaohsiung Medical University Hospital, Kaohsiung 80756, Taiwan; 12 Post-Baccalaureate Medicine, Kaohsiung Medical University, Kaohsiung 807, Taiwan

**Keywords:** Alzheimer’s dementia (AD), Clinical Dementia Rating (CDR), ganglion cell layer (GCL), inner plexiform layer (IPL), macular thickness, MMSE (Mini-Mental State Examination), nerve fiber layer (NFL), optical coherence tomography (OCT)

## Abstract

This retrospective case-control study aimed to investigate associations between disease severity of Alzheimer’s dementia (AD) and macular thickness. Data of patients with AD who were under medication (n = 192) between 2013 and 2020, as well as an age- and sex-matched control group (n = 200) with normal cognitive function, were included. AD patients were divided into subgroups according to scores of the Mini-Mental State Examination (MMSE) and Clinical Dementia Rating (CDR). Macular thickness was analyzed via the Early Treatment Diabetic Retinopathy Study (ETDRS) grid map. AD patients had significant reductions in full macula layers, including inner circle, outer inferior area, and outer nasal area of the macula. Similar retinal thinning was noted in ganglion cells and inner plexiform layers. Advanced AD patients (MMSE score < 18 or CDR ≥ 1) showed more advanced reduction of macular thickness than the AD group (CDR = 0.5 or MMSE ≥ 18), indicating that severe cognitive impairment was associated with thinner macular thickness. Advanced AD is associated with significant macula thinning in full retina and inner plexiform layers, especially at the inner circle of the macula. Macular thickness may be a useful biomarker of AD disease severity. Retinal imaging may be a non-invasive, low-cost surrogate for AD.

## 1. Introduction

Alzheimer’s dementia (AD) is a progressive neurodegenerative disorder that is the most common cause of dementia [1]. Currently, at least 44 million people are estimated to live with dementia worldwide, which is predicted to more than triple by 2050 as the population ages [2]. A definitive diagnosis of AD is based on amyloid β and tau pathology determined by invasive procedures [3].

Brain imaging, including magnetic resonance imaging (MRI) and positron emission tomography (PET), cerebrospinal fluid (CSF) biomarkers such as amyloid beta (Aβ) and tau, and serologic testing, including genetic markers, are reported to have diagnostic value [4]. However, routine use of these measures in clinical practice has limitations, including high cost, invasiveness, and limited sensitivity and specificity. Thus, AD remains primarily a clinical diagnosis. Currently, AD is diagnosed mainly by characteristic medical history, clinical neurological presentation, and cognitive examinations. However, cognitive assessment of AD using the Mini-Mental State Examination (MMSE) score and Clinical Dementia Rating (CDR) score is time-consuming and reaching diagnostic accuracy is difficult, even by experts [4].

The retina is an integral part of the central nervous system, and the concept of the retina as a surrogate of the brain has received increasing interest in recent years [5]. Retinal spectral-domain optical coherence tomography (OCT) scanning is a non-invasive, widely available imaging test used by ophthalmologists. It demonstrates retinal imaging at the histological level and enables additional reproducible dimensions of the retinal layer along with resolution of 1–2 microns. Two of the most performed OCT scans are the macular scan, which detects retinal pathologies, and the optic nerve head (ONH) scan of the retinal nerve fiber layer (RNFL). Since the retinal ganglion cell layer (GCL) and the ONH RNFL contain the neuronal body and axons connecting photoreceptors with visual pathways in the brain, degenerative changes in the brain have been reported to be detected by OCT scans. OCT complies with a wide variety of sustained studies released in prior years, revealing that cognitive impairment was related to decreased thickness of RNFL, GCL, and full macula layers [6]. Therefore, OCT has great potential to serve as a disease biomarker for AD [7].

The primary aim of the present study was to analyze the associations between the disease severity of Alzheimer’s dementia and macular thickness through retinal OCT images. We aimed to identify the decisive retinal layer and sectors of the retina for detection of AD, and we also correlated the results with clinical cognitive exams, including the MMSE and CDR scores. The study also demonstrates the possibility that macular OCT may address several clinically pertinent challenges in dealing with AD, including disease screening and the evaluation of AD disease severity.

## 2. Materials and Methods

### 2.1. Study Design and Data Sources

This retrospective case-control study extracted data from the hospital electronic medical records of Kaohsiung Medical University Hospital Database (KMUHRD), which includes various patient medical records from one medical center, two regional hospitals, and one local hospital in southern Taiwan. The KMUHRD data used in this study included patient data of outpatient visits, inpatient hospitalization, retinal spectral-domain OCT imaging and reports, and patient neuropsychological examination reports.

### 2.2. Ethical Considerations

The Institutional Review Board (IRB) and Ethics Committee of Kaohsiung Medical University Hospital reviewed and approved the study protocol, which also adheres to the Declaration of Helsinki. Since this study was retrospective in design and used existing patient information that was deidentified, the IRB also waived patients’ informed consent.

### 2.3. Study Sample

Patients diagnosed with AD based on ICD-9-CM and ICD-10 diagnosis codes and who received prescribed medication by neurologists between 2013 and 2020 in one medical center and one regional hospital of the KMUHRD database were included. The cognitive examination was obtained from the department of Neurology. Patients with diabetic macular edema (DME), proliferative diabetic retinopathy (PDR), glaucoma, optic neuropathy, and ocular disease that may lead to significant changes in retinal thickness were excluded.

**Figure 1:**
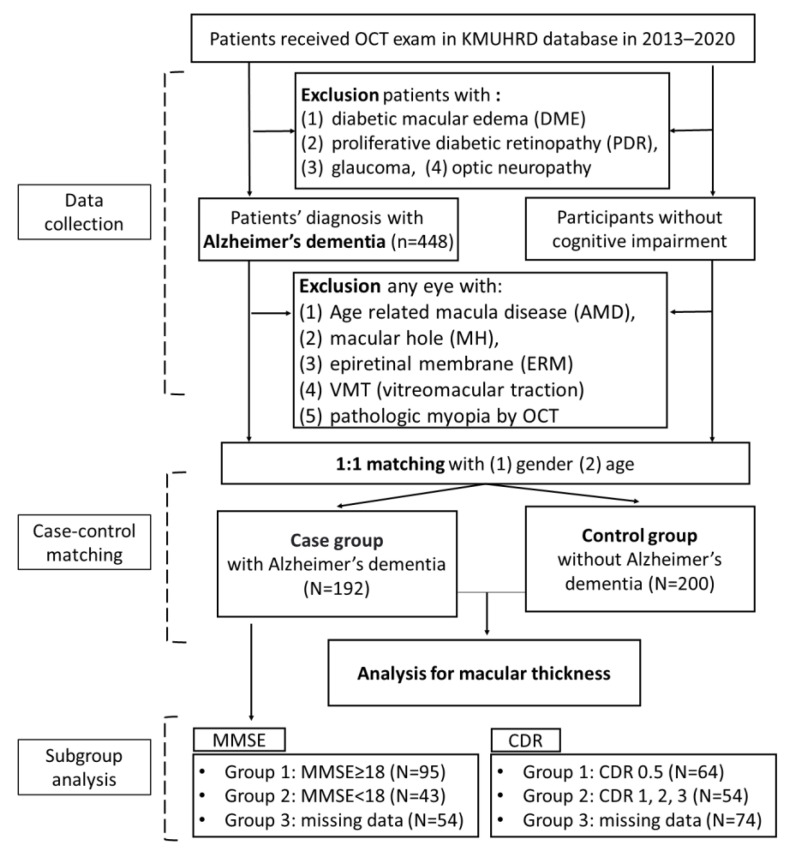
The algorithm of study design. OCT: optical coherence tomography; N: numbers of patients; KMUHRD: Kaohsiung Medical University Hospital Database; MMSE: Mini-Mental State Examination; CDR: Clinical Dementia Rating.

The included patients had records of OCT exams at ophthalmic visits, as well as OCT images taken close to the date of AD diagnosis within the time interval of 1–2 years. OCT images were generated by the Spectralis OCT (Heidelberg Engineering, Heidelberg, Germany). Initial quality control was performed by experienced ophthalmologists and technicians during the clinical OCT examination, to filter out images with low resolution or improper format as well as eyes with age-related macular disease (AMD), macular holes (MH), VMT (vitreomacular traction), and epiretinal membrane (ERM), even chorioretinal atrophy or prominent posterior staphyloma. Precise matching of patients’ age (by birth year) and sex was done to generate the comparison group (control group) of non-AD patients. Patients receiving OCT exams between 2013 and 2020, filtered by the exclusion criteria cited above, were included. Of these patients, under initial matching with sex and age and exclusion of outliers, 175 patients had AD under medication and 188 patients had no cognitive impairment. Figure 1 shows the study sample inclusion and exclusion algorithm of the study.

### 2.4. Measurements

**Figure 2:**
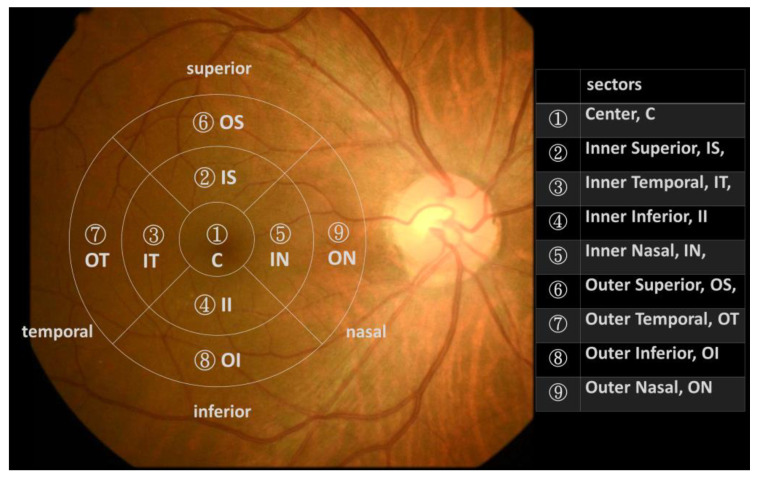
The adapted Early Treatment Diabetic Retinopathy Study (ETDRS) grid map.

The data augmentation procedure was performed by exporting the macular thickness map into different retinal layers, including full layers of macula, nerve fiber layer, ganglion cell layer, and inner plexiform layer, in the total four categories of thickness map. The entire thickness map was exported into the Early Treatment Diabetic Retinopathy Study (ETDRS) grid map, which was divided into nine sectors (one 1-millimeter-diameter circle at the center, four inner-quarter annuluses between the 1- and 3-millimeter-diameter circles, and four outer-quarter annuluses between the 3- and 6-millimeter diameter circles), as previously described [8]. Figure 2 showed the ETDRS grid map adapted from previous studies [9,10], and abbreviations and numbers were employed to represent the nine regions of macular thickness map in the present study.

### 2.5. Subgroup Analysis

Patients with AD were then divided into subgroups according to MMSE scores and CDR scores (Figure 1). MMSE is an exam of cognitive function, including orientation, concentration, attention, verbal memory, naming, and visuospatial skills. The exam is based on a total score of 30 points, and the better cognitive function is related to a higher score. As for CDR, it is rated according to six domains of cognitive function, including memory, orientation, judgment, problem solving, community affairs, home and hobbies, and personal care. The result is integrated into a 5-point scale as follows: 0, no impairment; 0.5, questionable impairment; 1, mild impairment; 2, moderate impairment; and 3, severe impairment. Given that no standardized MMSE cutoff values for dementia staging were available, we followed previous studies and classified AD patients into mild cognitive impairment (MMSE score ≥ 18) and moderate or severe impaired cognitive function (MMSE < 18) [11,12]. Because staging was based on CDR scores, patients were classified into one group with more severe cognitive impairment (CDR ≥ 1) and one group with mild cognitive impairment (CDR < 1). For those AD patients without MMSE or CDR records in the database, another group was classified as the group with missing data. The characteristics of age and sex among the three subgroups based on MMSE scores or CDR scores are presented in Appendix A, Table A1. Specifically, 87 patients who had MMSE scores ≥ 18, 37 patients with MMSE scores < 18, and 51 patients with missing MMSE data were recorded. In the CDR-based group, 58 patients with CDR = 0.5, 48 patients with CDR ≥ 1, and 69 patients with missing data were reported. Multivariable linear regression models were used later to analyze the associations between macular thickness and MMSE as well as CDR.

### 2.6. Statistical Analysis

Frequencies were used to present the proportions of demographic characteristics. Chi-square tests were used to compare differences between categorical variables. We first did the hypothesis testing for normality for the measures of ETDRS grid macular thickness using the Anderson-Darling test [13], which is one of the common approaches for the normality test, and the results supported the assumptions of normal distribution. We then used independent *t*-tests or ANOVA tests to compare continuous variables between groups. Multivariable linear regression models were used to compare the differences in ETDRS grid macular thickness between groups (AD versus controls, and different severity level of dementia based on MMSE and CDR) while controlling for age and sex. A two-tailed *p* < 0.05 was considered significant in all statistical tests. We further used the Bonferroni approach to calculate adjusted *p*-values among multiple site comparisons to handle multiple testing concerns [14]. All statistical operations were performed using SAS version 9.4 (SAS Analytics, Cary, NC, USA).

## 3. Results

### 3.1. Demographic Characteristics of the Participants

Table 1 shows the baseline demographic characteristics of age (based on birth year) and sex between matched AD patients (N = 175) and the comparison group (N = 188). No statistically significant differences were noted between these two groups. For AD patients, the mean age was 77.144 years with 65.14% older than 75 years, and 61.14% were female. For the non-AD comparison group, the mean age was 76.76 years with 62.23% older than 75 years, and 61.70% were female. MMSE and CDR scores among AD patients are also presented in Table 1.

### 3.2. Association between Macular Thickness and Alzheimer’s Dementia

**Figure 3:**
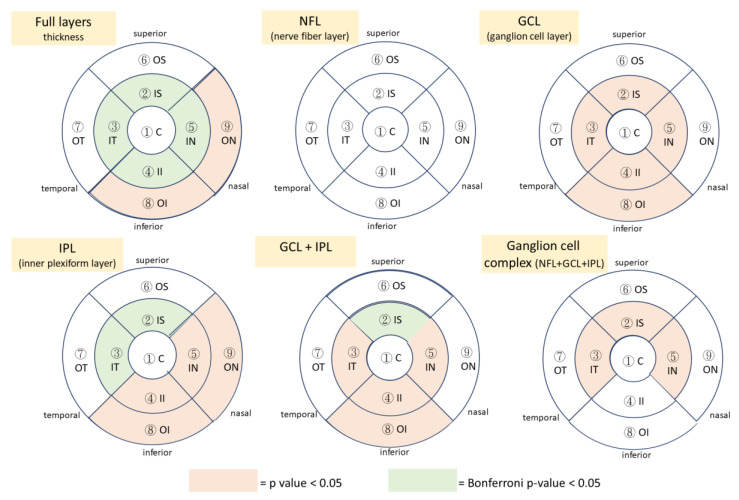
In the ETDRS grid map, the color-coded sectors showed significant decreased macular thickness in different retina layers in Alzheimer’s dementia. C: center; IS: inner superior; IT: inner temporal; II: inner inferior; IN: inner nasal; OS: outer superior; OT: outer temporal; OI: outer inferior; ON: outer nasal.

As shown in Table 2, retinal thickness was thinner in patients with AD in comparison to the control group with normal cognitive function. In the full macula layers, significant differences were observed between the groups in the inner superior (case: 322.90 ± 16.43 μm, control 329.00 ± 14.88 μm; beta coefficient: −7.03; *p*-value < 0.001), inner temporal area (case: 311.30 ± 15.89 μm, control 315.80 ± 14.76 μm; beta coefficient: −5.32; *p*-value = 0.005), inner inferior area (case: 319.00 ± 17.12 μm, control 324.50 ± 16.02 μm; beta coefficient: −7.00; *p*-value = 0.001), inner nasal area (case: 326.50 ± 17.62 μm, control 331.70 ± 16.58 μm; beta coefficient: −6.55; *p*-value = 0.004), outer inferior area (case: 272.60 ± 14.30 μm, control 2276.30 ± 12.56 μm; beta coefficient: −3.80; *p*-value = 0.011), and outer nasal area (case: 298.90 ± 15.84 μm, control 302.20 ± 14.56 μm; beta coefficient: −4.49; *p*-value = 0.043). The results were similar in the inner plexiform layer (IPL) and ganglion cell layer (GCL), which showed significantly decreased macular thickness in AD, especially in the inner circle part and outer inferior area in the ETDRS grid. However, when the data were defined solely on the NFL layer, no significant differences were noted in macular thickness between the AD group and the control group. Thus, when we converted the statistical results into plots of the ETDRS grid map, the area marked with a star represented a significant reduction of macular thickness in different retinal layers in AD (Figure 3).

When any one of the single layers, including NFL, GCL, and IPL, was statistically integrated to another one, or when the three single layers were all statistically combined, the results were similar, presenting significantly thinner thickness in the inner part of the macula in AD than in the control group. In addition, according to the Bonferroni approach to calculate adjusted *p*-values, the significant differences were noted in the inner area of macula in full thickness, IPL layer, and in GCL + IPL layer.

### 3.3. Associations between Macular Thickness and Severity of Dementia via Subgroup Analysis Stratified by MMSE and CDR Scores

In the initial results of MMSE subgroup analysis, significant differences were found in macular thickness in between-group comparisons, but the results were not consistent (Appendix B). After multivariable linear regression, when compared to the control group, group 2, with MMSE scores < 18, showed no significant reduction in any layer of macula thickness, and group 1, with MMSE ≥ 18, showed significantly thinner full macular thickness in the inner superior region (−6.00 µm (95%CI: −9.83, −2.17), Bonferronip-value 0.021) (Table 3). In the missing data group, the macular thickness was thinner than that in the control group in full layers of the retina, NFL, GCL, or IPL, and in almost every region of the macula.

**Figure 4:**
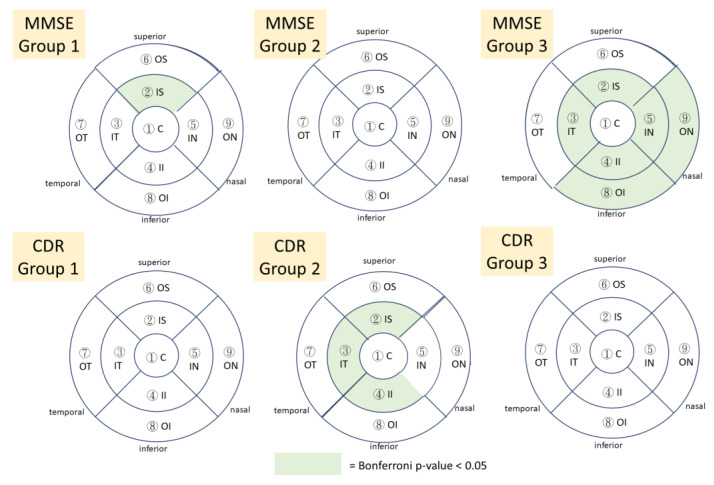
In the ETDRS grid map, the color-coded sectors showed significant decreased full retina thickness in different groups of neuropsychological severity in Alzheimer’s dementia. C: center; IS: inner superior; IT: inner temporal; II: inner inferior; IN: inner nasal; OS: outer superior; OT: outer temporal; OI: outer inferior; ON: outer nasal; MMSE: Mini-Mental State Examination, CDR: Clinical Dementia Rating.

Regarding CDR scores, the initial results showed no significant differences in between-group comparisons (Appendix B). After multivariable linear regression according to the Bonferroni approach to calculate adjusted *p*-values, in group 2, with CDR scores ≥ 1, significant reduction was observed in the macular thickness compared to that of the control group. The full layer of the retina revealed the most prominent results, especially in the inner superior region (−8.13 µm (95%CI: −12.81, −3.46), Bonferroni *p*-value = 0.006), inner temporal region (−7.34 µm (95%CI: −11.91, −2.78), Bonferroni *p*-value 0.015), and inner inferior region (−8.58 µm (95%CI: −13.48, −3.68), Bonferroni *p*-value = 0.006) (Table 4), and the results were similar in the inner temporal region of the IPL and ganglion cell complex. Interestingly, no significantly thinner macular thickness was found in the NFL layer. In group 1, with CDR < 1, macula thickness was generally decreased but no significant difference was noted via the Bonferroni approach. Macular thickness in the missing data group was thinner when compared to that of the control group, but the greater amount of macular thickness reduction was noted in group 2, with CDR score ≥ 1, rather than in the missing data group. Therefore, these results indicated that the advanced stage of dementia was associated with thinner macular thickness. The association between ETDRS macular thickness of the full retina layer and different groups of neuropsychological severity was presented in Figure 4.

## 4. Discussion

The present study showed that retinal thickness was thinner in patients with AD in comparison to that of people without AD. Significant differences were found in the thickness of full macular layers and IPL thickness, but not in NFL thickness. In addition, major differences were found at the inner sectors of the macula in the ETDRS grid map.

The retina shares similarities with the brain, and the concept of the retina as an exceptionally convenient window by which to assess the neuronal and vascular changes in the central nervous system (CNS) started decades ago. Various retinal changes in AD have been investigated in the last three decades. In 1986, Hinton et al. discovered in postmortem histological research that AD patients possessed prevalent axonal degeneration, decline in a variety of retinal ganglion cells (RCGs), and decline in the thickness of the retinal nerve fiber layer (RNFL) compared to those of age-matched controls [15]. This finding was further supported by electrophysiological research revealing unusual patterns in the electroretinogram as feasible documentation of RGC degeneration in AD [16,17]. Despite several inconsistencies, most studies revealed alterations in visual function, including visual acuity, contrast sensitivity, and color perception, as well as structural change such as a decrease in RNFL and GCL thickness and the pattern of ERG amplitude, a reduction of optic nerve fibers, and an increase of glial reactivity and cell loss in AD retinas. Several animal studies on the analysis of the OCT images showed a statistically significant thinning of the nerve fiber layer in AD mouse retinas compared to wild type controls, though there were some inconsistent findings in different reports. The inconsistency might be due to the young age of the tested animals or may suggest an initial inflammatory response that may lead to cell death and the consequent reduction in thickness later on [18,19,20]. Through the advance of ocular imaging technology, the current OCT technology allows high-resolution in vivo cross-sectional images as well as quantifiable and reproducible calculations of the macula and the possibility of ocular biomarkers for systemic diseases, including AD.

Results of the present study were compatible with previous reports demonstrating thinning of the retina, although in variable sectors [21,22,23,24,25,26,27]. Considering the pathological change of the retina in AD shows the deposition of amyloid β plaques in the retinal ganglion complex (RGC), which is a combination of nerve fibers, ganglion cells, and inner plexiform layers, RGC is the major structure affected in AD patients. Due to centrifugal distribution, there is more RGC in the inner part of the retina, which helps to explain the result showing change of full layer thickness, IPL, and GCL + IPL in the inner circle of macula related to thinner thickness due to AD. In our study, AD patients tended to have thinner thickness of full layer thickness, IPL, and GC-IPL, rather than solely NFL, and the result was similar to several reports [28,29]. The proportion GC-IPL is approximately 50% in RGC, whose cell bodies are over 10 to 20 times the diameter of their axons [29], and we hypothesized that GC-IPL might be more vulnerable to the damage in Alzheimer’s dementia (AD). In addition, though reduction of RNFL thickness in AD was reported [23], the evidence of dynamic changes of NFL and GC-IPL during AD progression was never reported [30]. A recently published convolutional neural network (CNN) to detect symptomatic Alzheimer’s dementia (AD) using a combination of multimodal retinal images and patient data also showed that GC-IPL maps were the most useful single inputs for prediction [31].

It is highly expected that changes in retinal thickness of AD patients correlates with the severity of AD. Currently, the relationship between macular thickness and the severity of cognitive function impairment is still not well established. A systematic review supported the presence of RNFL thinning and mild cognitive impairment (MCI), particularly amnestic MCI, though less remarkable than the associations with AD [32]. The present study included only AD patients who had received treatment, but not those with MCI. We categorized the AD patients into mild AD and moderate-to-severe AD based on MMSE scores and CDR grading. After multivariable linear regression, the MMSE scores correlated positively with macular thickness, and the association between CDR grading and macular thickness was much more significant. The significantly changed areas were at the inner circle of the macula, compatible with general analysis of AD patients versus controls. Variable sensitivity and specificity of MMSE scores may be due to patients’ age, sex, or education status [33], while for CDR scores, the sensitivity and specificity were reported to be as high as 87% and 99% [34], respectively. Thus, CDR scores may be a more objective and consistent parameter for evaluating the clinical cognitive condition. Since patients in the present study were missing certain MMSE or CDR data, the whole missing data group presented similar findings as those with AD. We speculated that patients in the missing data group might the more severe AD patients who could not complete the psychiatric and cognitive examinations. In other words, our results might support a possible association between AD and retinal thinning, which would be very helpful in monitoring the disease course or even treatment response. Integrated with brain imaging and biomarker and neuropsychological assessment, the eye could potentially improve AD risk assessment, detection, and monitoring [35].

This study has several limitations. First, it was a retrospective study, and OCT images taken around the time of AD diagnosis were retrieved, suggesting there may be a gap between the diagnosis or disease severity and the images taken. Some patients might not visit neurology department and ophthalmology department around the same time, and this was the possible reason leading to the gap in the retrospective study. Retrospective design also limits generalization of results to other populations and does not allow the ruling out of selection bias. Second, nearly 40% of cognitive examinations were missing from patients’ records at the time of switching from paper to electronic medical records or were simply not done. We are planning to conduct a further prospective longitudinal study where the patients may receive OCT imaging in a timelier manner during the initial diagnosis of AD, which will allow associations between the trend of macular thickness and the change of MMSE or CDR scores within several years to be detected prospectively. However, it should be noted that the present study included large case numbers, and the other systemic or ocular diseases were excluded or matched before analysis.

## 5. Conclusions

In conclusion, significant retinal thinning was noted in the retinas of AD patients, especially in full retinal layers and inner plexiform layers at the inner circle of the macula. Results of this study support that macular thickness may be a useful biomarker of the disease severity of AD. Retinal images may be non-invasive, low-cost surrogates for AD.

## Figures and Tables

**Table 1:** General characteristics of the participants. N: numbers of patients; y/o: years-old, MMSE: Mini-Mental State Examination, CDR: Clinical Dementia Rating, SD: standard deviation; 95% CI: 95% confidence interval; *p*-value < 0.05 is considered as statistically significant.

	Alzheimer’s Dementia (AD)	Control Group	
	N, %/Mean ± SD	N, %/Mean ± SD	*p*-Value
N	N = 175	N = 188	
Average Age (y/o)	77.14 ± 10.51	76.76 ± 10.46	0.725
<65	16 (9.14%)	16 (8.51%)	0.751
65~74	45 (25.71%)	55 (29.26%)	
≥75	114 (65.14%)	117 (62.23%)	
Gender			
Male	68 (38.86%)	72 (38.30%)	0.913
Female	107 (61.14%)	116(61.7%)	
Average MMSE Score	20.60 ± 5.49		
MMSE, Category			
≥18	87 (49.71%)		
<18	37 (21.14%)		
No Data	51 (29.14%)		
CDR, Category			
0.5	58 (33.14%)		
1	37 (21.14%)		
2,3	11 (6.29%)		
No Data	69 (39.43%)		

**Table 2:** Characteristics of ETDRS grid macular thickness in Alzheimer’s dementia (AD) and control group. NFL: nerve fiber layer; GCL: ganglion cell layer; IPL: inner plexiform layer; GCC: ganglion cell complex (NFL + GCL + IPL); N: numbers of patients; SD: standard deviation; 95% CI: 95% confidence interval; *p*-value < 0.05 is considered as statistically significant (*), and the bold numbers mean statistically significance with *p*-value < 0.05.

	Alzheimer’s Dementia (AD)	Control		Multivariable Linear Regression ^a^		
	N,%/Mean ± SD	N,%/Mean ± SD	*p*-Value	β (95% CI)	*p*-Value	Bonferroni *p*-Value
N	N = 175	N = 188				
Full thickness, μm						
central	257.70 ± 20.94	259.90 ± 21.25	0.329	−1.88 (−6.24, 2.48)	0.316	1.000
inner superior	**322.90 ± 16.43**	**329.00 ± 14.88**	<0.001 *	**−7.03 (−10.67, −3.39)**	<0.001 *	0.002 *
inner temporal	**311.30 ± 15.89**	**315.80 ± 14.76**	0.005 *	**−5.32** **(−8.68, −1.96)**	0.005 *	0.042 *
inner inferior	**319.00 ± 17.12**	**324.50 ± 16.02**	0.002 *	**−7.00** **(−10.71, −3.28)**	0.001 *	0.013 *
inner nasal	**326.50 ± 17.62**	**331.70 ± 16.58**	0.004 *	**−6.55 (−10.28, −2.83)**	0.004 *	0.032 *
outer superior	283.60 ± 14.72	284.60 ± 12.99	0.520	−2.24 (−5.28, 0.81)	0.549	1.000
outer temporal	269.90 ± 13.48	270.90 ± 11.76	0.459	−1.80 (−4.53, 0.93)	0.476	1.000
outer inferior	272.60 ± 14.30	276.30 ± 12.56	0.010 *	−3.80 (−7.06, −0.54)	0.011 *	0.096
outer nasal	298.90 ± 15.84	302.20 ± 14.56	0.039 *	−4.49 (−7.85, −1.12)	0.043 *	0.386
NFL thickness, μm						
central	11.59 ± 2.37	11.79 ± 2.47	0.423	−0.06 (−0.58, 0.46)	0.426	1.000
inner superior	25.26 ± 4.32	25.62 ± 3.98	0.409	−0.43 (−1.34, 0.48)	0.411	1.000
inner temporal	18.66 ± 1.96	18.46 ± 1.62	0.293	0.21 (−0.15, 0.57)	0.313	1.000
inner inferior	24.43 ± 4.14	24.52 ± 3.67	0.832	−0.19 (−0.99, 0.62)	0.853	1.000
inner nasal	21.63 ± 3.01	21.66 ± 2.59	0.918	−0.03 (−0.61, 0.55)	0.914	1.000
outer superior	38.99 ± 6.79	39.23 ± 5.94	0.720	−0.38 (−1.75, 0.98)	0.725	1.000
outer temporal	21.01 ± 2.61	20.64 ± 1.72	0.123	0.34 (−0.10, 0.78)	0.127	1.000
outer inferior	38.48 ± 8.30	38.48 ± 7.58	0.999	−0.03 (−1.68, 1.62)	0.962	1.000
outer nasal	48.99 ± 8.73	49.28 ± 8.29	0.747	−0.58 (−2.36, 1.19)	0.771	1.000
GCL thickness, μm						
central	13.84 ± 4.47	13.79 ± 4.33	0.909	0.58 (−0.51, 1.67)	0.892	1.000
inner superior	46.86 ± 5.83	48.70 ± 6.66	0.005 *	−2.40 (−3.81, −0.99)	0.006 *	0.052
inner temporal	41.38 ± 6.35	42.77 ± 6.58	0.041 *	−1.40 (−2.76, −0.03)	0.044 *	0.397
inner inferior	45.42 ± 6.31	47.04 ± 6.60	0.017 *	−1.92 (−3.39, −0.46)	0.018 *	0.160
inner nasal	45.86 ± 6.58	47.54 ± 7.55	0.025 *	−1.89 (−3.36, −0.42)	0.026 *	0.237
outer superior	31.55 ± 4.41	32.00 ± 3.68	0.298	−0.84 (−1.70, 0.02)	0.310	1.000
outer temporal	32.35 ± 4.84	32.36 ± 4.25	0.988	−0.29 (−1.28, 0.70)	0.985	1.000
outer inferior	30.02 ± 4.12	30.97 ± 3.71	0.021 *	−0.74 (−1.63, 0.16)	0.022 *	0.197
outer nasal	35.30 ± 4.11	35.96 ± 4.10	0.124	−0.81 (−1.67, 0.06)	0.133	1.000
IPL thickness, μm						
central	19.11 ± 3.82	19.34 ± 3.54	0.558	0.05 (−0.76, 0.86)	0.563	1.000
inner superior	**37.01 ± 3.70**	**38.24 ± 4.20**	0.003 *	**−1.55 (−2.44, −0.67)**	0.003 *	0.030 *
inner temporal	**37.47 ± 4.30**	**38.77 ± 4.25**	0.004 *	**−1.38 (−2.27, −0.49)**	0.004 *	0.037 *
inner inferior	36.42 ± 4.01	37.38 ± 4.15	0.027 *	−1.09 (−1.99, −0.18)	0.028 *	0.250
inner nasal	38.16 ± 4.05	39.31 ± 4.82	0.014 *	−1.33 (−2.27, −0.38)	0.016 *	0.140
outer superior	25.77 ± 2.98	25.98 ± 2.71	0.465	−0.45 (−1.05, 0.15)	0.495	1.000
outer temporal	29.91 ± 3.07	30.16 ± 2.91	0.424	−0.43 (−1.07, 0.20)	0.448	1.000
outer inferior	24.51 ± 2.87	25.26 ± 2.62	0.010 *	−0.67 (−1.29, −0.06)	0.010 *	0.092
outer nasal	27.01 ± 3.05	27.64 ± 2.99	0.047 *	−0.69 (−1.31, −0.07)	0.051	0.460
NFL + GCL thickness, μm						
central	25.43 ± 6.36	25.58 ± 6.47	0.823	0.52 (−1.01, 2.05)	0.837	1.000
inner superior	72.11 ± 8.90	74.32 ± 9.37	0.022 *	−2.83 (−4.87, −0.79)	0.025 *	0.221
inner temporal	60.03 ± 6.67	61.23 ± 6.77	0.092	−1.19 (−2.64, 0.27)	0.097	0.877
inner inferior	69.85 ± 9.34	71.56 ± 9.35	0.082	−2.11 (−4.19, −0.03)	0.087	0.782
inner nasal	67.49 ± 8.49	69.20 ± 9.15	0.066	−1.92 (−3.77, −0.07)	0.070	0.633
outer superior	70.54 ± 9.58	71.23 ± 8.32	0.466	−1.22 (−3.16, 0.72)	0.478	1.000
outer temporal	53.36 ± 6.11	53.01 ± 5.05	0.549	0.05 (−1.17, 1.27)	0.544	1.000
outer inferior	68.50 ± 10.92	69.45 ± 9.89	0.383	−0.77 (−3.00, 1.46)	0.409	1.000
outer nasal	84.29 ± 10.54	85.24 ± 10.32	0.385	−1.39 (−3.63, 0.85)	0.410	1.000
GCL + IPL thickness, μm						
central	32.95 ± 8.04	33.12 ± 7.53	0.832	0.63 (−1.21, 2.47)	0.844	1.000
inner superior	**83.86 ± 9.03**	**86.95 ± 10.38**	0.003 *	**−3.95 (−6.17, −1.74)**	0.003 *	0.026 *
inner temporal	78.85 ± 10.10	81.54 ± 10.31	0.013 *	−2.77 (−4.93, −0.62)	0.013 *	0.117
inner inferior	81.84 ± 9.92	84.42 ± 10.35	0.016 *	−3.01 (−5.30, −0.72)	0.016 *	0.147
inner nasal	84.02 ± 10.12	86.85 ± 11.82	0.015 *	−3.22 (−5.53, −0.90)	0.016 *	0.142
outer superior	57.32 ± 7.14	57.98 ± 6.19	0.343	−1.29 (−2.71, 0.13)	0.364	1.000
outer temporal	62.26 ± 7.63	62.52 ± 6.57	0.731	−0.72 (−2.27, 0.82)	0.759	1.000
outer inferior	54.53 ± 6.77	56.23 ± 6.14	0.012 *	−1.41 (−2.88, 0.06)	0.013 *	0.117
outer nasal	62.30 ± 6.80	63.60 ± 6.88	0.072	−1.50 (−2.93, −0.07)	0.078	0.698
NFL + IPL thickness, μm						
central	30.70 ± 5.85	31.13 ± 5.71	0.478	−0.01 (−1.28, 1.26)	0.482	1.000
inner superior	62.26 ± 6.91	63.86 ± 7.13	0.031 *	−1.98 (−3.54, −0.43)	0.034 *	0.304
inner temporal	56.13 ± 4.80	57.22 ± 4.60	0.027 *	−1.17 (−2.17, −0.17)	0.028 *	0.248
inner inferior	60.86 ± 7.27	61.90 ± 6.99	0.165	−1.27 (−2.81, 0.27)	0.175	1.000
inner nasal	59.79 ± 6.23	60.97 ± 6.51	0.079	−1.36 (−2.71, 0.00)	0.084	0.752
outer superior	64.75 ± 8.36	65.21 ± 7.44	0.581	−0.84 (−2.55, 0.88)	0.597	1.000
outer temporal	50.91 ± 4.60	50.80 ± 3.82	0.803	−0.09 (−1.00, 0.81)	0.803	1.000
outer inferior	62.99 ± 9.97	63.74 ± 8.86	0.448	−0.70 (−2.69, 1.29)	0.476	1.000
outer nasal	75.99 ± 9.89	76.91 ± 9.38	0.363	−1.28 (−3.32, 0.76)	0.386	1.000
NFL + GCL + IPL thickness, μm						
central	44.54 ± 9.96	44.91 ± 9.68	0.714	0.57 (−1.72, 2.85)	0.725	1.000
inner superior	109.10 ± 11.93	112.60 ± 12.98	0.009 *	−4.38 (−7.19, −1.58)	0.010 *	0.089
inner temporal	97.51 ± 10.33	99.99 ± 10.45	0.023 *	−2.56 (−4.79, −0.34)	0.024 *	0.219
inner inferior	106.30 ± 12.87	108.90 ± 13.00	0.050	−3.20 (−6.09, −0.31)	0.053	0.477
inner nasal	105.70 ± 12.02	108.50 ± 13.38	0.033 *	−3.25 (−5.94, −0.56)	0.035 *	0.317
outer superior	96.31 ± 11.75	97.21 ± 10.33	0.436	−1.67 (−4.07, 0.73)	0.453	1.000
outer temporal	83.27 ± 8.78	83.16 ± 7.34	0.903	−0.38 (−2.15, 1.39)	0.888	1.000
outer inferior	93.01 ± 13.05	94.71 ± 11.66	0.189	−1.44 (−4.12, 1.24)	0.204	1.000
outer nasal	111.30 ± 12.36	112.90 ± 12.13	0.218	−2.09 (−4.72, 0.55)	0.236	1.000

Note: ^a^: Age and sex were controlled and adjusted in the multiple variable regression models.

**Table 3:** ETDRS grid macular thickness stratified by MMSE scores. MMSE: Mini-Mental State Examination; NFL: nerve fiber layer; GCL: ganglion cell layer; IPL: inner plexiform layer; GCC: ganglion cell complex (NFL + GCL + IPL); N: numbers of patients; SD: standard deviation; 95% CI: 95% confidence interval; *p*-value is < 0.05 considered as statistically significant (*), and the bold numbers mean statistically significance with *p*-value < 0.05.

	Multivariable Linear Regression								
	Group 1 vs. Control			Group 2 vs. Control			Group 3 vs. Control		
	β (95% CI)	*p*-Value	Bonferroni *p*-Value	β (95% CI)	*p*-Value	Bonferroni *p*-Value	β (95% CI)	*p*-Value	Bonferroni *p*-Value
N	N = 87			N = 37			N = 51		
Full, μm									
C	−1.45 (−6.70, 3.80)	0.587	1.000	−2.45 (−10.01, 5.10)	0.523	1.000	−2.87 (−9.09, 3.36)	0.365	1.000
IS	**−6.00 (−9.83, −2.17)**	0.002 *	0.021 *	−3.31 (−8.54, 1.92)	0.214	1.000	**−8.23 (−12.79, −3.66)**	<0.001 *	0.004 *
IT	−3.41 (−7.07, 0.26)	0.068	0.616	−2.70 (−7.82, 2.42)	0.300	1.000	**−7.32 (−11.81, −2.83)**	0.002 *	0.012 *
II	−4.62 (−8.66, −0.58)	0.025 *	0.225	−0.74 (−6.20, 4.73)	0.791	1.000	**−10.03 (−14.71, −5.36)**	<0.001 *	0.001 *
IN	−5.10 (−9.35, −0.85)	0.019 *	0.171	−0.34 (−6.03, 5.35)	0.907	1.000	**−8.43 (−13.25, −3.62)**	<0.001 *	0.006 *
OS	−1.47 (−4.81, 1.87)	0.387	1.000	3.69 (−0.97, 8.34)	0.120	1.000	−3.16 (−7.38, 1.06)	0.141	1.000
OT	−1.12 (−4.12, 1.88)	0.464	1.000	1.84 (−2.37, 6.04)	0.390	1.000	−2.55 (−6.38, 1.27)	0.190	1.000
OI	−2.96 (−6.14, 0.22)	0.068	0.616	−0.39 (−4.99, 4.21)	0.867	1.000	**−6.91 (−10.94, −2.89)**	0.001 *	0.006 *
ON	−2.00 (−5.74, 1.73)	0.292	1.000	−0.15 (−5.28, 4.99)	0.955	1.000	**−7.38 (−11.83, −2.93)**	0.001 *	0.010 *
NFL, μm									
C	−0.32 (−0.92–0.29)	0.301	1.000	−0.19 (−1.05, 0.68)	0.672	1.000	0.04 (−0.72, 0.79)	0.920	1.000
IS	0.05 (−1.01–1.10)	0.927	1.000	−0.39 (−1.79, 1.02)	0.589	1.000	−1.03 (−2.31, 0.24)	0.112	1.000
IT	−0.10 (−0.50–0.29)	0.614	1.000	0.51 (−0.07, 1.09)	0.086	0.774	0.42 (−0.13, 0.97)	0.131	1.000
II	−0.28 (−1.23–0.66)	0.555	1.000	1.38 (0.08, 2.69)	0.038	0.344	−0.73 (−1.86, 0.40)	0.207	1.000
IN	0.01 (−0.68–0.70)	0.976	1.000	0.48 (−0.44, 1.40)	0.307	1.000	−0.46 (−1.26, 0.33)	0.251	1.000
OS	0.11 (−1.42–1.63)	0.892	1.000	1.87 (−0.29, 4.02)	0.090	0.812	−2.33 (−4.28, −0.38)	0.019 *	0.175
OT	0.29 (−0.17–0.75)	0.221	1.000	0.82 (0.18, 1.46)	0.013	0.114	0.13 (−0.57, 0.83)	0.718	1.000
OI	0.11 (−1.80–2.01)	0.914	1.000	2.33 (−0.55, 5.22)	0.113	1.000	−1.70 (−4.02, 0.61)	0.148	1.000
ON	0.71 (−1.37–2.78)	0.503	1.000	2.49 (−0.52, 5.50)	0.104	0.935	**−3.79 (−6.39, −1.19)**	0.004 *	0.040 *
GCL, μm									
C	0.05 (−1.06–1.15)	0.934	1.000	0.29 (−1.29, 1.87)	0.719	1.000	−0.08 (−1.40, 1.25)	0.907	1.000
IS	−1.57 (−3.13–0.00)	0.049	0.445	−0.13 (−2.41, 2.15)	0.908	1.000	**−3.35 (−5.35, −1.35)**	0.001 *	0.010 *
IT	−0.34 (−1.92–1.23)	0.666	1.000	−0.94 (−3.18, 1.29)	0.406	1.000	**−3.32 (−5.32, −1.32)**	0.001 *	0.011 *
II	−1.20 (−2.76–0.36)	0.132	1.000	−0.05 (−2.32, 2.22)	0.966	1.000	**−3.35 (−5.31, −1.38)**	<0.001 *	0.008 *
IN	−1.51 (−3.29–0.26)	0.095	0.853	0.44 (−2.09, 2.98)	0.731	1.000	**−3.23 (−5.42, −1.04)**	0.004 *	0.036 *
OS	−0.26 (−1.22–0.71)	0.602	1.000	1.14 (−0.19, 2.47)	0.092	0.825	**−1.82 (−3.02, −0.63)**	0.003 *	0.026 *
OT	0.16 (−0.91–1.23)	0.767	1.000	0.94 (−0.63, 2.51)	0.241	1.000	−0.94 (−2.36, 0.48)	0.195	1.000
OI	−0.82 (−1.79–0.14)	0.095	0.855	−1.02 (−2.38, 0.34)	0.141	1.000	−1.09 (−2.24, 0.07)	0.065	0.582
ON	−0.44 (−1.42–0.55)	0.382	1.000	0.06 (−1.36, 1.48)	0.933	1.000	−1.47 (−2.76, −0.18)	0.026 *	0.230
IPL, μm									
C	−0.16 (−1.08–0.77)	0.740	1.000	−0.31 (−1.59, 0.98)	0.639	1.000	−0.23 (−1.32, 0.87)	0.682	1.000
IS	−1.23 (−2.22–−0.24)	0.015	0.139	−0.45 (−1.86, 0.95)	0.524	1.000	−1.65 (−2.89, −0.41)	0.009 *	0.084
IT	−0.93 (−1.95–0.08)	0.072	0.651	−1.04 (−2.47, 0.39)	0.153	1.000	**−1.90 (−3.20, −0.59)**	0.005 *	0.041 *
II	−0.75 (−1.74–0.24)	0.135	1.000	−0.09 (−1.48, 1.30)	0.902	1.000	**−1.76 (−2.99, −0.53)**	0.005 *	0.048 *
IN	−1.00 (−2.13–0.14)	0.084	0.760	−0.53 (−2.12, 1.06)	0.511	1.000	−1.67 (−3.08, −0.26)	0.021 *	0.186
OS	−0.07 (−0.77–0.63)	0.844	1.000	0.85 (−0.11, 1.82)	0.084	0.753	−1.17 (−2.00, −0.33)	0.006 *	0.058
OT	−0.14 (−0.85–0.56)	0.692	1.000	0.42 (−0.63, 1.48)	0.429	1.000	−0.84 (−1.78, 0.10)	0.081	0.725
OI	−0.59 (−1.26–0.08)	0.082	0.736	−0.51 (−1.45, 0.44)	0.292	1.000	−1.16 (−2.01, −0.31)	0.008 *	0.068
ON	−0.37 (−1.11–0.36)	0.315	1.000	−0.14 (−1.18, 0.90)	0.785	1.000	−1.35 (−2.29, −0.40)	0.006 *	0.050
NFL + GCL,									
C	−0.27 (−1.88–1.34)	0.740	1.000	0.10 (−2.21, 2.42)	0.930	1.000	−0.04 (−2.01, 1.93)	0.968	1.000
IS	−1.52 (−3.81–0.78)	0.194	1.000	−0.52 (−3.77, 2.73)	0.753	1.000	**−4.38 (−7.28, −1.49)**	0.003 *	0.029 *
IT	−0.45 (−2.07–1.18)	0.590	1.000	−0.44 (−2.78, 1.91)	0.715	1.000	−2.90 (−5.00, −0.79)	0.007 *	0.065
II	−1.48 (−3.74–0.78)	0.198	1.000	1.33 (−1.89, 4.56)	0.416	1.000	**−4.07 (−6.84, −1.30)**	0.004 *	0.037 *
IN	−1.50 (−3.72–0.72)	0.183	1.000	0.92 (−2.19, 4.03)	0.560	1.000	−3.69 (−6.37, −1.02)	0.007 *	0.062
OS	−0.15 (−2.23–1.93)	0.887	1.000	3.01 (0.01, 6.01)	0.049	0.445	**−4.15 (−6.91, −1.40)**	0.003 *	0.030 *
OT	0.45 (−0.83–1.72)	0.491	1.000	1.75 (−0.14, 3.65)	0.069	0.624	−0.81 (−2.59, 0.97)	0.371	1.000
OI	−0.72 (−3.24–1.80)	0.576	1.000	1.31 (−2.41, 5.03)	0.488	1.000	−2.79 (−5.82, 0.24)	0.071	0.638
ON	0.27 (−2.22–2.75)	0.833	1.000	2.55 (−1.15, 6.25)	0.175	1.000	**−5.26 (−8.48, −2.05)**	0.001 *	0.013 *
GCL + IPL,									
C	−0.11 (−2.06–1.84)	0.912	1.000	−0.02 (−2.76, 2.73)	0.990	1.000	−0.31 (−2.62, 2.01)	0.794	1.000
IS	−2.79 (−5.23–−0.36)	0.025 *	0.223	−0.59 (−4.10, 2.92)	0.741	1.000	**−5.00 (−8.07, −1.93)**	0.002 *	0.014 *
IT	−1.28 (−3.73–1.18)	0.307	1.000	−1.98 (−5.46, 1.49)	0.262	1.000	**−5.22 (−8.33, −2.10)**	0.001 *	0.010 *
II	−1.95 (−4.41–0.50)	0.119	1.000	−0.14 (−3.65, 3.38)	0.939	1.000	**−5.11 (−8.14, −2.07)**	0.001 *	0.010 *
IN	−2.51 (−5.30–0.27)	0.077	0.689	−0.09 (−4.03, 3.85)	0.965	1.000	**−4.90 (−8.30, −1.49)**	0.005 *	0.045 *
OS	−0.33 (−1.95–1.29)	0.692	1.000	2.00 (−0.24, 4.23)	0.080	0.716	**−2.99 (−4.93, −1.05)**	0.003 *	0.023 *
OT	0.02 (−1.63–1.67)	0.981	1.000	1.36 (−1.08, 3.80)	0.273	1.000	−1.77 (−3.98, 0.43)	0.114	1.000
OI	−1.41 (−3.00–0.17)	0.081	0.725	−1.53 (−3.75, 0.70)	0.178	1.000	−2.25 (−4.18, −0.31)	0.023 *	0.208
ON	−0.81 (−2.47–0.84)	0.335	1.000	−0.08 (−2.47, 2.30)	0.945	1.000	−2.82 (−4.96, −0.68)	0.010 *	0.090
NFL + IPL,									
C	−0.47 (−1.92–0.97)	0.518	1.000	−0.49 (−2.53, 1.55)	0.635	1.000	−0.19 (−1.95, 1.57)	0.832	1.000
IS	−1.18 (−2.96–0.60)	0.194	1.000	−0.84 (−3.28, 1.60)	0.497	1.000	−2.68 (−4.90, −0.47)	0.018 *	0.159
IT	−1.03 (−2.15–0.09)	0.071	0.637	−0.53 (−2.09, 1.03)	0.505	1.000	−1.47 (−2.93, −0.01)	0.048 *	0.433
II	−1.04 (−2.74–0.67)	0.233	1.000	1.30 (−1.08, 3.68)	0.284	1.000	−2.49 (−4.59, −0.39)	0.021 *	0.185
IN	−0.99 (−2.60–0.63)	0.231	1.000	−0.05 (−2.25, 2.14)	0.961	1.000	−2.13 (−4.06, −0.20)	0.031 *	0.275
OS	0.04 (−1.84–1.91)	0.971	1.000	2.72 (0.04, 5.40)	0.047	0.424	**−3.50 (−5.92, −1.07)**	0.005 *	0.044 *
OT	0.14 (−0.81–1.09)	0.765	1.000	1.24 (−0.19, 2.67)	0.089	0.805	−0.71 (−2.06, 0.65)	0.304	1.000
OI	−0.49 (−2.74–1.76)	0.670	1.000	1.83 (−1.53, 5.18)	0.284	1.000	−2.86 (−5.61, −0.12)	0.041 *	0.371
ON	0.33 (−1.96–2.62)	0.777	1.000	2.35 (−1.04, 5.74)	0.174	1.000	**−5.14 (−8.07, −2.20)**	<0.001 *	0.006 *
GCC, μm									
C	−0.43 (−2.89–2.03)	0.732	1.000	−0.20 (−3.69, 3.29)	0.909	1.000	−0.27 (−3.24, 2.70)	0.859	1.000
IS	−2.75 (−5.88–0.39)	0.086	0.776	−0.97 (−5.41, 3.46)	0.665	1.000	**−6.03 (−9.97, −2.09)**	0.003 *	0.025 *
IT	−1.38 (−3.88–1.12)	0.279	1.000	−1.47 (−5.03, 2.08)	0.414	1.000	**−4.79 (−8.00, −1.59)**	0.004 *	0.032 *
II	−2.23 (−5.36–0.89)	0.160	1.000	1.25 (−3.18, 5.68)	0.580	1.000	**−5.83 (−9.66, −2.00)**	0.003 *	0.027 *
IN	−2.50 (−5.72–0.72)	0.127	1.000	0.39 (−4.10, 4.88)	0.865	1.000	−5.36 (−9.24, −1.48)	0.007 *	0.062
OS	−0.22 (−2.81–2.37)	0.867	1.000	3.86 (0.14, 7.58)	0.042 *	0.377	**−5.32 (−8.68, −1.96)**	0.002 *	0.018 *
OT	0.31 (−1.54–2.15)	0.745	1.000	2.18 (−0.58, 4.93)	0.121	1.000	−1.65 (−4.19, 0.89)	0.203	1.000
OI	−1.31 (−4.29–1.68)	0.389	1.000	0.81 (−3.55, 5.17)	0.715	1.000	−3.95 (−7.57, −0.33)	0.032 *	0.292
ON	−0.11 (−3.00–2.79)	0.942	1.000	2.41 (−1.92, 6.74)	0.274	1.000	**−6.61 (−10.38, −2.84)**	<0.001 *	0.005 *

Note: Age and sex were controlled and adjusted in the multiple variable regression models.

**Table 4:** ETDRS grid macular thickness stratified by CDR scores. CDR: clinical dementia rating; NFL: nerve fiber layers; GCL: ganglion cell layer; IPL: inner plexiform layer; GCC: ganglion cell complex (NFL + GCL + IPL); N: numbers of patients; SD: standard deviation; 95% CI: 95% confidence interval; *p*-value < 0.05 is considered as statistically significant (*), and the bold numbers mean statistically significance with *p*-value < 0.05.

	Multivariable Linear Regression								
	Group 1 vs. Control			Group 2 vs. Control			Group 3 vs. Control		
	β (95% CI)	*p*-Value	Bonferroni *p*-Value	β (95% CI)	*p*-Value	Bonferroni *p*-Value	β (95% CI)	*p*-Value	Bonferroni *p*-Value
N	N = 58			N = 48			N = 69		
Full, μm									
C	−2.74 (−8.76, 3.29)	0.372	1.000	−4.68 (−11.24, 1.88)	0.161	1.000	0.06 (−5.68, 5.80)	0.983	1.000
IS	−4.99 (−9.38, −0.60)	0.026 *	0.235	**−8.13 (−12.81, −3.46)**	0.001 *	0.006 *	−5.48 (−9.61, −1.36)	0.009 *	0.084
IT	−2.22 (−6.42, 1.98)	0.299	1.000	**−7.34 (−11.91, −2.78)**	0.002 *	0.015 *	−4.15 (−8.18, −0.11)	0.044 *	0.398
II	−2.21 (−6.78, 2.36)	0.341	1.000	**−8.58 (−13.48, −3.68)**	0.001 *	0.006 *	−5.72 (−10.02, −1.42)	0.009 *	0.085
IN	−3.73 (−8.60, 1.15)	0.133	1.000	−7.11 (−12.20, −2.01)	0.007 *	0.059	−4.79 (−9.18, −0.40)	0.033 *	0.294
OS	−2.24 (−6.03, 1.55)	0.246	1.000	−0.16 (−4.36, 4.05)	0.942	1.000	−0.11 (−3.93, 3.72)	0.956	1.000
OT	−1.61 (−5.06, 1.83)	0.357	1.000	0.17 (−3.62, 3.96)	0.931	1.000	−1.07 (−4.48, 2.35)	0.539	1.000
OI	−3.54 (−7.29, 0.21)	0.064	0.577	−2.20 (−6.34, 1.93)	0.295	1.000	−4.57 (−8.10, −1.03)	0.012 *	0.104
ON	−2.21 (−6.51, 2.10)	0.314	1.000	−4.62 (−9.25, 0.00)	0.050	0.452	−2.96 (−6.97, 1.06)	0.149	1.000
NFL, μm									
C	−0.32 (−1.02–0.38)	0.366	1.000	−0.26 (−1.03, 0.51)	0.511	1.000	−0.05 (−0.73, 0.64)	0.896	1.000
IS	−0.29 (−1.46–0.88)	0.625	1.000	−0.71 (−1.99, 0.58)	0.280	1.000	−0.16 (−1.33, 1.01)	0.786	1.000
IT	−0.17 (−0.63–0.30)	0.478	1.000	0.38 (−0.13, 0.90)	0.145	1.000	0.35 (−0.14, 0.83)	0.159	1.000
II	0.56 (−0.49–1.62)	0.296	1.000	−0.53 (−1.64, 0.58)	0.346	1.000	−0.19 (−1.28, 0.91)	0.738	1.000
IN	0.25 (−0.54–1.04)	0.535	1.000	−0.41 (−1.20, 0.39)	0.316	1.000	0.01 (−0.73, 0.76)	0.970	1.000
OS	0.27 (−1.53–2.06)	0.769	1.000	−0.25 (−2.10, 1.60)	0.790	1.000	−0.63 (−2.43, 1.17)	0.490	1.000
OT	0.64 (−0.02–1.30)	0.059	0.528	0.23 (−0.32, 0.77)	0.418	1.000	0.20 (−0.32, 0.72)	0.446	1.000
OI	−0.45 (−2.80–1.90)	0.707	1.000	0.41 (−1.97, 2.80)	0.734	1.000	0.30 (−1.81, 2.41)	0.778	1.000
ON	1.14 (−1.42–3.70)	0.380	1.000	−1.81 (−4.37, 0.75)	0.166	1.000	−0.34 (−2.65, 1.98)	0.775	1.000
GCL, μm									
C	−0.23 (−1.47–1.00)	0.711	1.000	−0.03 (−1.39, 1.34)	0.969	1.000	0.36 (−0.90, 1.62)	0.572	1.000
IS	−0.92 (−2.76–0.91)	0.323	1.000	−2.49 (−4.54, −0.45)	0.017 *	0.155	−1.98 (−3.74, −0.23)	0.027 *	0.243
IT	0.10 (−1.73–1.93)	0.912	1.000	−2.34 (−4.30, −0.37)	0.020 *	0.179	−1.77 (−3.57, 0.04)	0.055	0.496
II	−0.60 (−2.42–1.23)	0.520	1.000	−2.05 (−4.03, −0.07)	0.043 *	0.383	−1.96 (−3.74, −0.18)	0.031 *	0.277
IN	−1.09 (−3.14–0.97)	0.300	1.000	−1.77 (−4.01, 0.47)	0.121	1.000	−1.89 (−3.88, 0.10)	0.063	0.569
OS	−0.49 (−1.59–0.60)	0.377	1.000	0.37 (−0.84, 1.59)	0.545	1.000	−0.85 (−1.92, 0.23)	0.121	1.000
OT	−0.33 (−1.59–0.92)	0.600	1.000	0.54 (−0.87, 1.94)	0.451	1.000	−0.01 (−1.27, 1.24)	0.983	1.000
OI	−1.46 (−2.62–−0.31)	0.014 *	0.122	−0.58 (−1.77, 0.62)	0.344	1.000	−0.73 (−1.73, 0.28)	0.156	1.000
ON	−0.71 (−1.88–0.46)	0.232	1.000	−0.67 (−1.96, 0.61)	0.302	1.000	−0.52 (−1.64, 0.60)	0.362	1.000
IPL, μm									
C	−0.53 (−1.58–0.52)	0.322	1.000	−0.27 (−1.39, 0.85)	0.636	1.000	0.08 (−0.94, 1.11)	0.873	1.000
IS	−0.68 (−1.83–0.46)	0.241	1.000	−1.60 (−2.84, −0.35)	0.012 *	0.110	−1.29 (−2.40, −0.18)	0.023 *	0.210
IT	−0.41 (−1.59–0.76)	0.487	1.000	−1.90 (−3.15, −0.66)	0.003 *	0.025 *	−1.44 (−2.61, −0.26)	0.017 *	0.154
II	−0.32 (−1.45–0.81)	0.578	1.000	−1.35 (−2.58, −0.11)	0.033 *	0.299	−1.04 (−2.15, 0.08)	0.068	0.610
IN	−0.71 (−2.01–0.59)	0.284	1.000	−1.35 (−2.76, 0.07)	0.063	0.569	−1.22 (−2.49, 0.05)	0.059	0.528
OS	−0.24 (−1.03–0.55)	0.551	1.000	0.46 (−0.43, 1.35)	0.312	1.000	−0.59 (−1.34, 0.17)	0.128	1.000
OT	−0.14 (−0.97–0.70)	0.745	1.000	−0.15 (−1.07, 0.76)	0.742	1.000	−0.35 (−1.19, 0.49)	0.417	1.000
OI	−0.90 (−1.70–−0.10)	0.028 *	0.255	−0.40 (−1.24, 0.44)	0.353	1.000	−0.84 (−1.57, −0.11)	0.025 *	0.221
ON	−0.41 (−1.28–0.46)	0.356	1.000	−0.71 (−1.66, 0.25)	0.146	1.000	−0.69 (−1.50, 0.12)	0.097	0.870
NFL + GCL,									
C	−0.55 (−2.37–1.27)	0.551	1.000	−0.28 (−2.30, 1.73)	0.781	1.000	0.32 (−1.52, 2.16)	0.734	1.000
IS	−1.21 (−3.86–1.43)	0.366	1.000	−3.20 (−6.15, −0.25)	0.034 *	0.303	−2.14 (−4.71, 0.42)	0.101	0.908
IT	−0.06 (−1.97–1.85)	0.947	1.000	−1.95 (−3.99, 0.09)	0.060	0.543	−1.42 (−3.31, 0.48)	0.141	1.000
II	−0.04 (−2.63–2.55)	0.978	1.000	−2.58 (−5.37, 0.21)	0.069	0.625	−2.15 (−4.73, 0.43)	0.103	0.924
IN	−0.84 (−3.38–1.71)	0.517	1.000	−2.18 (−4.91, 0.56)	0.119	1.000	−1.87 (−4.34, 0.59)	0.136	1.000
OS	−0.22 (−2.73–2.28)	0.860	1.000	0.13 (−2.46, 2.71)	0.924	1.000	−1.48 (−3.97, 1.01)	0.244	1.000
OT	0.30 (−1.29–1.89)	0.707	1.000	0.76 (−0.89, 2.41)	0.363	1.000	0.19 (−1.33, 1.71)	0.808	1.000
OI	−1.91 (−5.02–1.19)	0.226	1.000	−0.16 (−3.28, 2.95)	0.917	1.000	−0.42 (−3.13, 2.29)	0.759	1.000
ON	0.43 (−2.62–3.48)	0.780	1.000	−2.48 (−5.66, 0.69)	0.125	1.000	−0.86 (−3.73, 2.02)	0.558	1.000
GCL + IPL,									
C	−0.76 (−2.95–1.42)	0.493	1.000	−0.30 (−2.67, 2.08)	0.806	1.000	0.45 (−1.75, 2.64)	0.690	1.000
IS	−1.61 (−4.45–1.23)	0.266	1.000	−4.09 (−7.23, −0.95)	0.011 *	0.098	−3.27 (−5.99, −0.55)	0.019 *	0.167
IT	−0.31 (−3.15–2.53)	0.829	1.000	−4.24 (−7.28, −1.21)	0.006 *	0.057	−3.20 (−6.03, −0.38)	0.026 *	0.238
II	−0.92 (−3.75–1.92)	0.526	1.000	−3.40 (−6.49, −0.31)	0.031 *	0.282	−3.00 (−5.76, −0.24)	0.034 *	0.302
IN	−1.80 (−4.99–1.40)	0.270	1.000	−3.12 (−6.61, 0.38)	0.081	0.725	−3.11 (−6.20, −0.02)	0.048 *	0.436
OS	−0.73 (−2.57–1.10)	0.432	1.000	0.83 (−1.21, 2.87)	0.422	1.000	−1.43 (−3.20, 0.33)	0.110	0.991
OT	−0.47 (−2.41–1.47)	0.633	1.000	0.39 (−1.77, 2.54)	0.725	1.000	−0.36 (−2.32, 1.59)	0.717	1.000
OI	−2.36 (−4.26–−0.46)	0.015 *	0.135	−0.97 (−2.95, 1.00)	0.333	1.000	−1.57 (−3.25, 0.11)	0.067	0.605
ON	−1.12 (−3.08–0.84)	0.262	1.000	−1.38 (−3.55, 0.79)	0.211	1.000	−1.21 (−3.05, 0.64)	0.200	1.000
NFL + IPL,									
C	−0.85 (−2.50–0.80)	0.311	1.000	−0.53 (−2.31, 1.26)	0.562	1.000	0.04 (−1.58, 1.66)	0.963	1.000
IS	−0.98 (−2.99–1.04)	0.341	1.000	−2.30 (−4.52, −0.09)	0.042 *	0.376	−1.45 (−3.45, 0.54)	0.153	1.000
IT	−0.58 (−1.88–0.72)	0.378	1.000	−1.52 (−2.88, −0.16)	0.029 *	0.260	−1.09 (−2.41, 0.23)	0.106	0.950
II	0.24 (−1.67–2.16)	0.804	1.000	−1.88 (−3.95, 0.20)	0.076	0.683	−1.22 (−3.19, 0.75)	0.223	1.000
IN	−0.46 (−2.30–1.38)	0.622	1.000	−1.75 (−3.70, 0.19)	0.077	0.695	−1.21 (−2.98, 0.57)	0.181	1.000
OS	0.03 (−2.20–2.26)	0.979	1.000	0.21 (−2.11, 2.52)	0.859	1.000	−1.22 (−3.43, 1.00)	0.280	1.000
OT	0.50 (−0.73–1.73)	0.422	1.000	0.07 (−1.14, 1.28)	0.907	1.000	−0.15 (−1.30, 1.01)	0.804	1.000
OI	−1.35 (−4.12–1.43)	0.340	1.000	0.01 (−2.79, 2.82)	0.992	1.000	−0.54 (−3.01, 1.93)	0.669	1.000
ON	0.73 (−2.09–3.56)	0.609	1.000	−2.51 (−5.40, 0.38)	0.088	0.792	−1.02 (−3.65, 1.61)	0.444	1.000
GCC, μm									
C	−1.08 (−3.86–1.69)	0.443	1.000	−0.55 (−3.58, 2.48)	0.720	1.000	0.40 (−2.38, 3.18)	0.777	1.000
IS	−1.90 (−5.51–1.71)	0.302	1.000	−4.79 (−8.80, −0.79)	0.019 *	0.174	−3.43 (−6.94, 0.07)	0.055	0.491
IT	−0.48 (−3.39–2.43)	0.746	1.000	−3.86 (−6.95, −0.77)	0.015 *	0.131	−2.86 (−5.76, 0.05)	0.054	0.483
II	−0.36 (−3.92–3.21)	0.845	1.000	−3.93 (−7.79, −0.06)	0.046 *	0.417	−3.18 (−6.73, 0.36)	0.078	0.705
IN	−1.55 (−5.22–2.13)	0.408	1.000	−3.52 (−7.50, 0.46)	0.082	0.742	−3.10 (−6.65, 0.46)	0.088	0.788
OS	−0.46 (−3.55–2.62)	0.768	1.000	0.58 (−2.66, 3.83)	0.724	1.000	−2.07 (−5.11, 0.98)	0.183	1.000
OT	0.17 (−2.09–2.42)	0.885	1.000	0.61 (−1.78, 3.00)	0.615	1.000	−0.16 (−2.38, 2.06)	0.888	1.000
OI	−2.81 (−6.47–0.85)	0.132	1.000	−0.56 (−4.26, 3.13)	0.765	1.000	−1.26 (−4.47, 1.94)	0.438	1.000
ON	0.02 (−3.50–3.55)	0.989	1.000	−3.19 (−6.93, 0.56)	0.095	0.855	−1.54 (−4.90, 1.82)	0.367	1.000

Note: Age and sex were controlled and adjusted in the multiple variable regression models.

## Data Availability

Not applicable.

## Appendix A

**Table A1:** Characteristics of subgroup analysis stratified by MMSE and CDR scores. N: numbers of patients; y/o: years-old; MMSE: Mini-Mental State Examination, CDR: Clinical Dementia Rating; SD: standard deviation; 95% CI: 95% confidence interval; *p*-value < 0.05 is considered as statistically significant (*).

	MMSE Score				CDR Score			
	Group 1 (MMSE ≥ 18)	Group 2 (MMSE < 18)	Group 3 (Missing Data)		Group 1 (CDR = 0.5)	Group 2 (CDR = 1, 2)	Group 3 (Missing Data)	
	N, %/Mean ± SD	N, %/Mean ± SD	N, %/Mean ± SD	*p*-Value	N, %/Mean ± SD	N, %/Mean ± SD	N, %/Mean ± SD	*p*-Value
N	N = 87	N = 37	N = 51		N = 58	N = 48	N = 69	
Age (y/o)	75.97 ± 10.91	80.76 ± 6.76	76.53 ± 11.60	0.059	77.76 ± 9.66	77.15 ± 10.85	76.62 ± 11.07	0.834
Age, category								
<65	7 (8.05%)	1 (2.70%)	8 (15.69%)	0.004 *	4 (6.90%)	7 (14.58%)	5 (7.25%)	0.024 *
65~74	29 (33.33%)	3 (8.11%)	13 (25.49%)		13 (22.41%)	6 (12.50%)	26 (37.68%)	
≥75	51 (58.62%)	33 (89.19%)	30 (58.82%)		41 (70.69%)	35 (72.92%)	38 (55.07%)	
Gender								
Male	34 (39.08%)	14 (37.84%)	20 (39.22%)	0.990	23 (39.66%)	23 (47.92%)	22 (31.88%)	0.214
Female	53 (60.92%)	23 (62.16%)	31 (60.78%)		35 (60.34%)	25 (52.08%)	47 (68.12%)	

## Appendix B

**Table A2:** Between-group comparisons via subgroup analysis stratified by MMSE and CDR scores. N: numbers of patients; MMSE: Mini-Mental State Examination; CDR: Clinical Dementia Rating; NFL: nerve fiber layers; GCL: ganglion cell layer; IPL: inner plexiform layer; GCC: ganglion cell complex (NFL + GCL + IPL); SD: standard deviation; 95% CI: 95% confidence interval; *p*-value < 0.05 is considered as statistically significant (*).

	MMSE Group				CDR Group			
	Group 1 (MMSE ≥ 18)	Group 2 (MMSE < 18)	Group 3 (Missing Data)		Group 1 (CDR = 0.5)	Group 2 (CDR = 1, 2, 3)	Group 3 (Missing Data)	
	Mean ± SD	Mean ± SD	Mean ± SD	*p*-Value	Mean ± SD	Mean ± SD	Mean ± SD	*p*-Value
N	N = 87	N = 37	N = 51		N = 58	N = 48	N = 69	
Full, μm								
C	258.70 ± 20.90	256.20 ± 24.82	257.20 ± 18.12	0.810	257.00 ± 20.71	256.10 ± 21.46	259.40 ± 20.96	0.663
IS	323.40 ± 17.00	324.40 ± 15.89	320.90 ± 15.94	0.571	323.70 ± 17.46	321.00 ± 15.18	323.40 ± 16.49	0.646
IT	312.70 ± 15.61	311.60 ± 16.35	308.60 ± 16.00	0.338	313.30 ± 16.15	308.70 ± 15.15	311.40 ± 16.14	0.334
II	320.20 ± 17.82	322.00 ± 16.56	314.60 ± 15.71	0.081	321.90 ± 17.66	316.10 ± 15.86	318.50 ± 17.35	0.210
IN	327.00 ± 19.19	329.60 ± 17.46	323.40 ± 14.51	0.246	327.70 ± 19.95	324.90 ± 16.47	326.70 ± 16.45	0.715
OS	283.30 ± 13.83	287.40 ± 13.79	281.50 ± 16.49	0.162	282.10 ± 13.10	284.30 ± 14.50	284.40 ± 16.19	0.640
OT	269.90 ± 12.53	271.80 ± 13.48	268.40 ± 15.04	0.494	269.10 ± 12.89	271.20 ± 13.04	269.70 ± 14.36	0.719
OI	273.60 ± 13.21	274.90 ± 15.27	269.40 ± 15.10	0.146	272.50 ± 13.95	274.00 ± 14.89	271.80 ± 14.32	0.717
ON	300.50 ± 15.99	300.70 ± 15.03	294.90 ± 15.72	0.099	299.70 ± 15.98	297.30 ± 15.68	299.30 ± 15.99	0.724
NFL, μm								
C	11.51 ± 2.30	11.43 ± 2.48	11.84 ± 2.44	0.655	11.45 ± 2.20	11.58 ± 2.36	11.71 ± 2.54	0.827
IS	25.70 ± 4.48	25.14 ± 3.65	24.59 ± 4.47	0.339	25.31 ± 3.90	24.94 ± 4.09	25.43 ± 4.82	0.825
IT	18.34 ± 1.53	19.08 ± 1.99	18.88 ± 2.48	0.100	18.33 ± 1.70	18.92 ± 1.83	18.75 ± 2.23	0.268
II	24.30 ± 4.04	25.59 ± 4.23	23.82 ± 4.16	0.128	25.02 ± 3.85	24.00 ± 2.95	24.25 ± 4.99	0.404
IN	21.71 ± 3.15	22.03 ± 3.00	21.22 ± 2.77	0.436	21.90 ± 3.33	21.35 ± 2.37	21.61 ± 3.15	0.653
OS	39.34 ± 6.17	41.05 ± 6.48	36.88 ± 7.53	0.013 *	39.48 ± 6.52	38.79 ± 5.38	38.71 ± 7.87	0.795
OT	20.91 ± 2.00	21.57 ± 2.17	20.76 ± 3.63	0.324	21.31 ± 3.43	20.88 ± 1.79	20.84 ± 2.29	0.556
OI	38.69 ± 7.68	40.30 ± 10.51	36.80 ± 7.29	0.141	37.88 ± 9.43	38.75 ± 7.30	38.80 ± 8.02	0.798
ON	50.07 ± 7.96	51.24 ± 9.26	45.51 ± 8.74	0.002 *	50.31 ± 9.85	47.38 ± 6.99	49.00 ± 8.75	0.228
GCL, μm								
C	13.89 ± 4.48	13.89 ± 4.99	13.73 ± 4.13	0.977	13.52 ± 3.79	13.85 ± 4.22	14.10 ± 5.17	0.766
IS	47.25 ± 5.58	47.97 ± 5.67	45.37 ± 6.17	0.079	47.60 ± 5.49	46.04 ± 5.81	46.80 ± 6.11	0.389
IT	42.57 ± 6.15	41.16 ± 5.66	39.49 ± 6.78	0.021 *	42.71 ± 5.90	40.33 ± 5.27	40.99 ± 7.24	0.129
II	46.00 ± 6.11	46.35 ± 6.05	43.75 ± 6.63	0.076	46.28 ± 5.94	44.90 ± 5.64	45.06 ± 7.03	0.446
IN	46.18 ± 6.68	47.16 ± 6.07	44.35 ± 6.60	0.114	46.24 ± 6.22	45.63 ± 6.00	45.70 ± 7.30	0.862
OS	31.77 ± 4.22	32.95 ± 4.11	30.18 ± 4.62	0.011 *	31.43 ± 4.10	32.19 ± 4.68	31.22 ± 4.48	0.489
OT	32.56 ± 4.19	33.14 ± 5.03	31.43 ± 5.63	0.226	31.97 ± 4.30	32.81 ± 4.96	32.36 ± 5.20	0.671
OI	30.18 ± 4.09	29.81 ± 4.53	29.88 ± 3.90	0.866	29.45 ± 4.66	30.25 ± 4.15	30.33 ± 3.58	0.437
ON	35.57 ± 3.69	35.76 ± 3.90	34.49 ± 4.85	0.245	35.16 ± 3.90	35.10 ± 4.22	35.55 ± 4.26	0.805
IPL, μm								
C	19.22 ± 3.90	18.84 ± 4.09	19.12 ± 3.54	0.880	18.78 ± 3.76	19.13 ± 3.46	19.38 ± 4.13	0.679
IS	37.10 ± 3.78	37.32 ± 3.34	36.61 ± 3.83	0.632	37.43 ± 3.54	36.50 ± 3.43	37.00 ± 3.99	0.437
IT	37.94 ± 4.14	37.16 ± 4.05	36.90 ± 4.73	0.347	38.22 ± 4.11	36.81 ± 3.45	37.30 ± 4.92	0.224
II	36.74 ± 4.04	36.76 ± 3.70	35.65 ± 4.15	0.261	36.93 ± 3.69	35.94 ± 3.79	36.33 ± 4.41	0.437
IN	38.41 ± 4.26	38.24 ± 3.41	37.67 ± 4.15	0.576	38.47 ± 3.78	37.85 ± 3.73	38.12 ± 4.50	0.739
OS	25.94 ± 2.97	26.65 ± 2.85	24.82 ± 2.89	0.013 *	25.69 ± 2.72	26.33 ± 3.28	25.43 ± 2.96	0.270
OT	30.06 ± 2.56	30.35 ± 3.38	29.33 ± 3.58	0.252	29.97 ± 2.72	29.96 ± 2.93	29.83 ± 3.46	0.960
OI	24.69 ± 2.69	24.65 ± 2.82	24.10 ± 3.21	0.480	24.33 ± 3.02	24.77 ± 2.80	24.48 ± 2.82	0.729
ON	27.30 ± 2.77	27.30 ± 2.83	26.29 ± 3.57	0.142	27.17 ± 2.94	26.79 ± 3.32	27.01 ± 2.99	0.817
NFL + GCL								
C	25.39 ± 6.27	25.32 ± 6.98	25.57 ± 6.18	0.982	24.97 ± 5.51	25.44 ± 6.05	25.81 ± 7.26	0.759
IS	72.95 ± 8.82	73.11 ± 8.19	69.96 ± 9.33	0.121	72.91 ± 8.16	70.98 ± 8.81	72.23 ± 9.58	0.535
IT	60.92 ± 6.21	60.24 ± 6.27	58.37 ± 7.47	0.093	61.03 ± 6.34	59.25 ± 5.33	59.74 ± 7.68	0.351
II	70.30 ± 9.14	71.95 ± 9.26	67.57 ± 9.45	0.077	71.29 ± 8.78	68.90 ± 7.68	69.30 ± 10.74	0.349
IN	67.90 ± 8.84	69.19 ± 8.04	65.57 ± 7.99	0.117	68.14 ± 8.40	66.98 ± 7.31	67.30 ± 9.37	0.764
OS	71.11 ± 8.31	74.00 ± 8.91	67.06 ± 11.02	0.002 *	70.91 ± 9.20	70.98 ± 8.08	69.93 ± 10.86	0.792
OT	53.47 ± 4.87	54.70 ± 6.24	52.20 ± 7.65	0.161	53.28 ± 6.26	53.69 ± 5.55	53.20 ± 6.43	0.908
OI	68.87 ± 10.54	70.11 ± 13.18	66.69 ± 9.66	0.317	67.33 ± 12.83	69.00 ± 9.78	69.13 ± 9.96	0.609
ON	85.64 ± 9.20	87.00 ± 11.30	80.00 ± 11.05	0.002 *	85.47 ± 10.85	82.48 ± 9.13	84.55 ± 11.16	0.338
GCL + IPL								
C	33.10 ± 8.15	32.73 ± 8.83	32.84 ± 7.37	0.967	32.29 ± 7.30	32.98 ± 7.42	33.48 ± 9.05	0.712
IS	84.36 ± 9.03	85.30 ± 8.60	81.98 ± 9.19	0.182	85.03 ± 8.66	82.54 ± 8.77	83.80 ± 9.49	0.368
IT	80.52 ± 9.85	78.32 ± 9.28	76.39 ± 10.72	0.064	80.93 ± 9.51	77.15 ± 8.16	78.29 ± 11.55	0.133
II	82.74 ± 9.92	83.11 ± 9.43	79.39 ± 10.02	0.110	83.21 ± 9.38	80.83 ± 9.07	81.39 ± 10.90	0.422
IN	84.60 ± 10.58	85.41 ± 9.18	82.02 ± 9.84	0.227	84.71 ± 9.57	83.48 ± 9.46	83.81 ± 11.07	0.807
OS	57.71 ± 7.04	59.59 ± 6.88	55.00 ± 6.94	0.008 *	57.12 ± 6.68	58.52 ± 7.70	56.65 ± 7.10	0.368
OT	62.62 ± 6.50	63.49 ± 8.12	60.76 ± 8.88	0.212	61.93 ± 6.85	62.77 ± 7.55	62.19 ± 8.37	0.850
OI	54.87 ± 6.64	54.46 ± 7.09	53.98 ± 6.86	0.756	53.78 ± 7.46	55.02 ± 6.78	54.81 ± 6.17	0.582
ON	62.87 ± 6.18	63.05 ± 6.62	60.78 ± 7.77	0.165	62.33 ± 6.48	61.90 ± 7.40	62.57 ± 6.72	0.873
NFL + IPL								
C	30.72 ± 5.81	30.27 ± 6.26	30.96 ± 5.70	0.861	30.22 ± 5.57	30.71 ± 5.41	31.09 ± 6.40	0.712
IS	62.80 ± 7.20	62.46 ± 5.61	61.20 ± 7.26	0.413	62.74 ± 6.25	61.44 ± 6.40	62.43 ± 7.78	0.608
IT	56.29 ± 4.51	56.24 ± 4.26	55.78 ± 5.65	0.829	56.55 ± 4.68	55.73 ± 3.52	56.06 ± 5.64	0.673
II	61.03 ± 7.14	62.35 ± 6.99	59.47 ± 7.58	0.177	61.95 ± 6.56	59.94 ± 5.93	60.58 ± 8.55	0.339
IN	60.13 ± 6.73	60.27 ± 5.49	58.88 ± 5.85	0.462	60.36 ± 6.41	59.21 ± 5.13	59.72 ± 6.79	0.635
OS	65.29 ± 7.44	67.70 ± 7.88	61.71 ± 9.32	0.003 *	65.17 ± 7.91	65.13 ± 7.01	64.14 ± 9.58	0.741
OT	50.97 ± 3.47	51.92 ± 4.81	50.10 ± 5.91	0.185	51.28 ± 5.03	50.83 ± 3.57	50.67 ± 4.90	0.753
OI	63.38 ± 9.31	64.95 ± 12.07	60.90 ± 9.18	0.150	62.21 ± 11.32	63.52 ± 8.95	63.28 ± 9.52	0.761
ON	77.37 ± 8.68	78.54 ± 10.67	71.80 ± 10.15	0.001 *	77.48 ± 10.44	74.17 ± 8.28	76.01 ± 10.35	0.230
GCC								
C	44.61 ± 9.95	44.16 ± 10.87	44.69 ± 9.46	0.967	43.74 ± 9.02	44.56 ± 9.25	45.19 ± 11.20	0.719
IS	110.10 ± 12.07	110.40 ± 10.77	106.60 ± 12.31	0.191	110.30 ± 11.07	107.50 ± 11.54	109.20 ± 12.88	0.469
IT	98.86 ± 9.93	97.41 ± 9.55	95.27 ± 11.32	0.144	99.26 ± 9.89	96.06 ± 8.02	97.04 ± 11.94	0.255
II	107.00 ± 12.77	108.70 ± 12.46	103.20 ± 12.99	0.105	108.20 ± 12.01	104.80 ± 11.02	105.60 ± 14.61	0.351
IN	106.30 ± 12.75	107.40 ± 11.02	103.20 ± 11.25	0.209	106.60 ± 11.80	104.80 ± 10.66	105.40 ± 13.16	0.739
OS	97.06 ± 10.46	100.60 ± 11.04	91.88 ± 13.04	0.002 *	96.60 ± 11.19	97.31 ± 10.59	95.36 ± 13.02	0.662
OT	83.53 ± 7.07	85.05 ± 9.31	81.53 ± 10.71	0.165	83.24 ± 8.58	83.65 ± 8.02	83.03 ± 9.55	0.933
OI	93.56 ± 12.60	94.76 ± 15.19	90.78 ± 12.08	0.318	91.66 ± 15.12	93.77 ± 11.97	93.61 ± 11.96	0.630
ON	112.90 ± 10.65	114.30 ± 13.28	106.30 ± 13.15	0.002 *	112.60 ± 12.16	109.30 ± 11.36	111.60 ± 13.17	0.369
